# Paracentesis Tympani

**Published:** 1871-02

**Authors:** W. H. Fitch

**Affiliations:** Rockford, Illinois


### PARACENTÉSIS TYMPANI.

By W. H. FITCH, M.D., Rockford, Illinois.

I do not propose to write an article, at the present time, on the above theme, but simply to communicate the notes of a case in which deafness was cured by perforating the drum, and evacuating the acccumulations in the middle ear.

*Case.*—S. W., a farmer, *æt.* 37. On November 27, W. presented himself at my office, on account of almost complete deafness in his left ear. He stated that in March last, he took a severe cold in the head, since which time he has not been free from a troublesome nasal catarrh.

A few days after taking cold, he began to experience a peculiar sensation in both ears, attended with severe headache, chills, fever, loss of appetite, and general malaise.

Three days later, he was relieved of most of these symptoms, but observed in their stead, a continual roaring or buzzing in the left ear, together with a slight dulness of hearing. This latter symptom has increased, until, as before stated, there was almost total deafness, while the roaring remained about as at first, aggravated at times, however, when greatly fatigued or upon taking slight cold, so as often to interfere with sleep.

In the left ear, a watch could only be heard when placed on the organ; in the right ear, at the usual distance. Only the loudest tones of the voice could be distinguished when spoken in the ear.

The tuning-fork, placed on the head, was heard about equally in both ears.

An examination of the left external meatus, revealed, first, an accumulation of wax on the drum, which was easily washed away.

The manubrial plexus was somewhat injected. The manubrium mallie apparently drawn slightly inward and backward, while the membrana tympani was convex outward in the anterior and posterior segments. The lower two-thirds of the tympanum presented a dark, lustreless brown appearance, the upper third normal.

On the anterior segment was a small white cicatrix, the result of a former ulcer or perforation. Patient had once suffered, after an attack of measles, with running at the ears.

I at once diagnosed a serous accumulation within the tympanic cavity. I requested the patient to lie on the lounge a few moments, and then examined the drum in this position, and found that the line of fluid had also somewhat changed.

I next introduced the Eustachian Catheter, the smallest size, and applied the air douche. The air entered with great difficulty, and was attended with a bubbling rale. I may mention, that I had previously tried Politzer's method, but without results.

After this application, the hearing was somewhat improved, but the patient lived at a distance, and could not conveniently come daily to try the results of this method, so I concluded to evacuate the fluid at once, and, with a Myringtome, I perforated the tympanum in the posterior segment, causing little or no pain. Through the wound exuded a chocolate, half-jelly-like fluid in drops. I then applied the air douche again, and forced what I could of the remaining accumulation through the incision, in all, I should judge, over half a drachm.

The patient was at once relieved of the roaring in the ear, and the hearing much improved. Watch at 12 inches, and ordinary conversational tones easily heard.

Rhinoscopic examination revealed hyperemia of the mucous membrane of posterior nares, and narrowing of the entrance to the eustachian tube. I ordered an astringent powder to be snuffed up the nose, and some cotton wool to be kept in the ear a few days. Saw patient month afterwards. Hearing in both ears normal; nasal catarrh had much improved. Patient was satisfied with his condition.

As above indicated, my ringotomy consists in simple paracentésis of the membrana tympani, and I may mention in conclusion a few circumstances under which I have seen the operation performed at the clinics of Drs. Gruber and Politzer, at the K. K. Allgemeinen Krankenhaus in Vienna:

(1)—To open the way for operations on the deeper structures of the ear, such as (*a*) the breaking up of synechia between the tympanum and promontorium, or other structures, which have resisted the air douche; (*b*), tenotomy of the tensor tympani, not unfrequently indicated.

(2)—To give exit to accumulations in the internal ear, (*a*) extravastations of blood; (*b*), carious bone; (*c*), exudations after otitis media purulenta acuta, or (*d*), pus during the progress of this disease.

(3)—As an experimental operation, when neither by the eye or the history of the case are we able to come to a satisfactory diagnosis and logically institute other treatment, as when there exists too great tension of the tympanum, acting as an impediment to its normal vibrations. Hence, after the operation, I have seen the wound gape open as if it were before too small and tense for its place. The wound becomes filled with cicatricial tissue, and, in many cases, the deafness and other symptoms have been relieved.

Sometimes an improvement in the hearing occurred immediately after the operation and before the gap had been filled; at other times no improvement was observable until after cicitrization had taken place, and, in some instances, with the last named indications, no benefit was realized from the procedure. I might, perhaps, specify other conditions under which the operation would be justifiable, but enough have already been noticed to show that sufficient causes exist to render the operation one frequently indicated and often attended with the most pleasing results to the patient as well as surgeon. And I am fully convinced from my observations that hundreds of sufferers, who, having ears, hear not, might be forever rid of their infirmity if the profession generally better understood the indications for this little operation, and, recognizing the indications, would not hesitate to perform it.

